# Lokal fortgeschrittene oder oligometastasierte Blasenkarzinome – Stellenwert der Lokaltherapie von Primärtumor und Metastasen

**DOI:** 10.1007/s00120-021-01712-4

**Published:** 2021-11-26

**Authors:** Katharina Rebhan, Kilian M. Gust

**Affiliations:** Universitätsklinik für Urologie, Medizinische Universität Wien, Währinger Gürtel 18–20, 1090 Wien, Österreich

**Keywords:** Urothelkarzinom, Lymphknoten, Radikale Zystektomie, Radiotherapie, Chemotherapie, Urothelial cancer, Lymph nodes, Radical cystectomy, Radiation, Chemotherapy

## Abstract

**Hintergrund:**

Das muskelinvasive Blasenkarzinom stellt in seiner Behandlung eine besondere Herausforderung dar, da die Therapie mit signifikanten Nebenwirkungen und Komplikationsraten einhergeht, insbesondere bei Patienten mit relevanten Begleiterkrankungen. Im metastasierten Stadium besteht der Therapiezweck in der Palliation, wobei das Vorliegen einer Oligometastasierung eine gesonderte Rolle einnimmt. In diesem Stadium kann auch die Therapie des Primärtumors relevant sein, wenn die Metastasen neben einer systemischen Therapie ebenso lokal behandelt werden können – insbesondere auch in Hinblick auf die über die letzten Jahre die Therapielandschaft erweiternden neuen medikamentösen Möglichkeiten.

**Ziel der Arbeit:**

In diesem Reviewartikel sollen die Einflüsse einer definitiven Therapie des Primärtumors bei Patienten mit oligometastasiertem Urothelkarzinom der Harnblase dargelegt werden.

**Material und Methoden:**

Basierend auf einer nicht-systemischen Literaturrecherche soll ein Überblick über bestehende Ergebnisse zur Therapie des oligometastasierten Blasenkarzinoms in Hinblick auf den Einfluss der Therapie des Primärtumors geben, wobei die Daten meist auf retrospektiven Studien und Metaanalysen bestehen.

**Schlussfolgerung:**

Eine Lokaltherapie des Primärtumors im Rahmen eines multimodalen Therapiekonzepts kann bei selektionierten Patienten mit lymphogen metastasiertem und oligometastasiertem Blasenkarzinom einen positiven Einfluss auf Überleben, Lebensqualität und Vermeidung von Lokalkomplikationen nehmen, wobei für die Wahl der lokalen Therapie dieselben Kriterien angewendet werden sollten wie im nicht-metastasierten Stadium.

## Abstract (en)

**Background:**

Treatment of muscle invasive bladder cancer can be challenging since treatment is associated with significant side effects and complication rates—especially in patients who often present with relevant comorbidities. In the metastatic stage, the purpose of treatment is palliation, although the oligometastatic stage takes a distinct role. At this stage, treatment of the primary tumor can play a role, if metastasis can be treated locally in addition to systemic treatment, especially the evolving drug treatment landscape could also change long holding paradigms in the near future.

**Objectives:**

This review focuses on the influence of definitive treatment of the primary tumor in patients with oligometastatic urothelial bladder cancer.

**Materials and methods:**

Based on a literature search, the aim was to summarize data and give an overview on treatment of oligo-metastatic bladder cancer with focus on treatment of the primary tumor. Presented data derived mostly from retrospective studies and meta-analyses.

**Conclusion:**

Local treatment of the primary tumor in context of a multimodal therapy of lymph node metastatic or oligometastatic bladder cancer can have a positive influence on survival, quality of life and prevention of local complications in selected patients. The choice of local treatment should follow the same criteria as in non-metastatic bladder cancer.

## Hintergrund – Was sind die aktuellen Probleme?

Die Standardtherapie des muskelinvasiven Urothelkarzinoms der Blase (MIBC) ist die radikale Zystektomie (RZ).

Beim MIBC kann eine TMT für ältere und vorerkrankte Patienten eine Alternative zur RZ sein

Darüber hinaus kann eine neoadjuvante Cisplatin-haltige Chemotherapie das Gesamtüberleben verbessern [1]. Da die RZ jedoch mit einer hohen perioperativen Morbidität und Mortalität einhergeht, sind in den letzten Jahrzehnten organerhaltende Therapieoptionen entwickelt worden [2]. Patienten, die eine RZ erhalten, sind in der Regel jünger und in besserem Allgemeinzustand, jedoch kann eine trimodale Therapie (TMT), bestehend aus vollständiger transurethraler Resektion der Blase (TURB), einer Cisplatin-basierten Kombinationschemotherapie sowie der Bestrahlung der Blase insbesondere für ältere und vorerkrankte Patienten eine Alternative mit vergleichbarem onkologischen Ergebnis sein [3–5].

Um Patienten mit fortgeschrittenem oder oligometastasiertem MIBC bestmöglich zu behandeln, sollten zur Entscheidungsfindung folgende Fragen beantwortet werden:

1. Wird eine zytoreduktive RZ oder eine hochdosierte Strahlentherapie nach einer systemischen Chemotherapie das Überleben beeinflussen?
2. Kann eine metastasengezielte Therapie das Überleben verlängern (Metastasektomie oder stereotaktische Bestrahlung)?

## Blasenerhaltende Therapie vs. radikale Zystektomie

Da es bisher keine randomisiert kontrollierten Studien gibt, welche TMT mit RZ vergleichen, basieren Therapieempfehlungen auf retrospektiven Studien, systematischen Reviews und Metaanalysen [6]. Eine aktuelle Analyse von Patienten mit MIBC konnte keinen signifikanten Unterschied im Gesamtüberleben (64,3 vs. 70,7 Monate; *p* = 0,84) oder im krankheitsfreien 5‑Jahres-Überleben (73,2 vs. 76,6 Monate; *p* = 0,49) feststellen [7]. Eine etwas ältere Metaanalyse an über 12.000 Patienten zeigte ebenfalls keinen Unterschied im Gesamtüberleben, progressionsfreiem oder krankheitsspezifischem Überleben nach 5 und 10 Jahren [2].

In Bezug auf die Lebensqualität zeigte die TMT sorgfältig selektierter Patienten einen möglichen Vorteil des Organerhalts unter Berücksichtigung der Lebensqualität verglichen mit RZ (zusätzliche 1,6 „Qualitätsbereinigte Lebensjahre“; [8]). Optimale Kandidaten für eine TMT sind jene, bei denen eine vollständige TURB durchgeführt werden kann, keine Hydronephrose vorliegt und die Funktion der Blase nicht eingeschränkt ist [6]. Blasenerhaltende operative Therapieansätze können bei hochselektionierten Patienten in Erwägung gezogen werden, wenn ein adäquater tumorfreier Absetzungsrand bei Erhalten der Funktionalität gewährleistet werden kann [3].

Patienten mit metastatischen Absiedlungen in mehreren Organen dürften hinsichtlich des Gesamtüberlebens nicht von einer konsolidierenden RZ profitieren. Die Indikation zur RZ bei diesen Patienten hat lokale Tumorkontrolle zum Therapieziel [1, 9]. Allerdings ist das krankheitsspezifische Überleben bei Patienten mit einem cM1-Tumorstadium mit jenem mit cN2‑3 oder cT3-T4, cN+, M0 vergleichbar [10].

## Therapie beim lymphogenen und fernmetastasierten Urothelkarzinom der Blase

Eine induktive Chemotherapie ermöglicht es jene Patienten zu selektieren, deren Tumorbiologie eine geringere Aggressivität zeigt [11]. Das Ansprechen der Lymphknotenmetastasen auf eine Chemotherapie kann als Surrogatparameter hinsichtlich des Gesamtüberlebens in der Klinik genutzt werden [12].

Für ein chirurgisches Vorgehen nach einer Chemotherapie sprechen mehrere Gründe: zum einen ist eine systemische, alleinige Chemotherapie selten kurativ, zum anderen entstehen etwa 75 % der Rezidive an der Stelle des anfänglichen Ansprechens auf eine Chemotherapie. Mittels Resektion und histologischer Aufarbeitung des Gewebes kann außerdem das Therapieansprechen genauer eingeschätzt werden. Bei der Patientenselektion sollte auf eine resezierbare Metastasenlast, ein Ansprechen auf die Chemotherapie und einen guten Performancestatus geachtet werden [10].

Patienten, welche eine zytoreduktive oder konsolidierende RZ bei solitären Metastasen erhielten, zeigen einen krankheitsspezifischen Überlebensvorteil von 18 Monaten (Hazard Ratio [HR] 2,62: *p* = 0,02). In dieser Studie an 43 Patienten waren 56 % von postoperativen Komplikationen betroffen und es kam zu einem perioperativen Todesfall [13]. Einschränkend muss auf die geringe Größe der Studie hingewiesen werden und die Tatsache, dass es noch keine Daten aus der Zeit der Erhaltungstherapie mit Avelumab gibt.

Eine große Kohortenstudie schloss 3753 Patienten mit metastasiertem Blasenkarzinom (supraregionale Lymphknoten, viszerale oder Knochenmetastasen) ein. Das mediane Gesamtüberleben verlängerte sich signifikant (14,9 vs. 9,8 Monate; HR 0,56; *p* < 0,001), wenn Patienten eine intensive lokale Therapie (Strahlentherapie mit ≥ 50 Gy oder RZ) erhielten. Verglichen wurde hierbei mit einer konservativen lokalen Therapie (keine lokale Therapie, TURB allein oder Strahlentherapie mit < 50 Gy). Außerdem zeigte die Subgruppenanalyse einen noch größeren Überlebensvorteil, wenn die Patienten eine konsolidierende RZ nach Chemotherapie erhielten (17,7 Monate; [11]).

Durch eine lokale Therapie können blasenkarzinomtypische Symptome verhindert werden

Für eine lokale Therapie beim oligometastasierten Blasenkarzinom spricht, dass vom Primärtumorgewebe weniger metastasenfördernde Faktoren sezerniert werden dürften [14]. Des Weiteren können durch eine lokale Therapie blasenkarzinomtypische Symptome verhindert werden, welche ansonsten die Lebensqualität stark reduzieren können (z. B. Makrohämaturie, Schmerzen, Nierenversagen). Aktuell werden klinische Studien beim lokal fortgeschrittenen und oligometastasierten Blasenkarzinom durchgeführt (Tab. 1).

**Table 1:** 

Studien ID	Phase	Population	Intervention	*n*	Primärer Endpunkt
NCT03529890 (RACE IT)	2	Lokal fortgeschrittenes Urothelkarzinom der Blase	Neoadjuvant Nivolumab + Radiatio vor RZ mit pelviner Lymphadenektomie	33	Abschluss der Behandlung
NCT04047693	2	MIBC und lokal fortgeschrittenes Urothelkarzinom der Blase	Neoadjuvant dosisdicht MVAC (Methotrexat, Vinblastin, Doxorubicin + Cisplatin) + G-CSF	32	Vollständige Remissionsrate
NCT02989584	1/2	Phase 2: resezierbares Urothelkarzinom cT2-4a; N0/X; M0	Neoadjuvant Atezolizumab, Gemcitabin + Cisplatin vor RZ	54	Sicherheit
NCT04724928 (EFFORT-MIBC)	2	Nicht-metastasiertes, oligometastasiertes oder metastasiertes MIBC	Neoadjuvant Chemotherapie + RZ oder TMT ± metastasengerichtete Therapie oder Immuntherapie	156	2‑Jahres-Gesamtüberleben
NCT03601455	1/2	Nicht resezierbar, lokal fortgeschritten oder metastasiertes Urothelkarzinom der Blase	Radiatio + Durvalumab	13	Sicherheit, progressionsfreies Überleben

*RZ* radikale Zystektomie, *TMT* trimodale Therapie, *MIBC* muskelinvasives Urothelkarzinom der Blase, *TURB* transurethrale Resektion der Blase, *MVAC* Methotrexat/Vinblastin/Adriamycin/Cisplatin, *G‑CSF* granulozytenkoloniestimulierender Faktor

## Rationale für metastasengezielte Therapie

Bereits 1982 wurde das Prinzip der Metastasektomie bei Patienten mit Blasenkarzinom und solitären Lungenmetastasen evaluiert. Jene 6 Patienten, welche eine Thorakotomie mit Wedge-Resektion erhielten, zeigten ein medianes Überleben von 5 Jahren. Verglichen mit der damals durchschnittlichen Überlebenszeit des metastasierten Blasenkarzinoms (3 Monate) war dies wegweisend für weitere Studien zur Metastasektomie [15]. Das Prinzip der Metastasektomie beruht auf dem Konzept, dass mittels der Entfernung des Primärtumors bereits jene Wachstumsfaktoren entzogen werden, welche eine Fernmetastasierung begünstigen und somit durch die anschließende Metastasektomie eine Chance auf Heilung besteht. Ein Ansprechen auf eine zytoreduktive Therapie von Metastasen konnte in soliden Tumoren wie z. B. dem Nierenzellkarzinom nachgewiesen werden [16].

Gute Ansprechraten zeigte eine weitere Studie an 32 Patienten, welche sich einer pulmonalen Metastasektomie unterzogen (5-Jahres-Gesamtüberleben von 50 %, progressionsfreies Überleben von 26 %). Besonders Patienten mit kleinen pulmonalen Metastasen (< 3 cm) profitieren mit einem progressionsfreiem Überleben von 65 % von dieser Therapie [9]. Patienten, welche eine systemische Chemotherapie vor der Metastasektomie erhalten, zeigen bessere Überlebensraten verglichen mit alleiniger Metastasektomie [17]. Das mediane Gesamtüberleben liegt nach einer Metastasektomie beim Blasenkarzinom zwischen 29 und 42 Monaten [17–19].

Patienten mit kleinen pulmonalen Metastasen profitieren von einer pulmonalen Metastasektomie

Eine retrospektive Studie untersuchte 13 Patienten, welche eine stereotaktische Radiotherapie oder eine konformale dreidimensional (3D-)geplante Bestrahlung ihrer Metastasen erhielten. Insgesamt 21 Läsionen in Lymphknoten, Knochen oder der Lunge wurden dabei mit 20–36 Gy aufgeteilt auf 3–10 Fraktionen bestrahlt. 3 der 13 oligometastasierten Patienten waren nach 25 Monaten ohne Krankheitsnachweiß. Somit kann ein oligometastasiertes Blasenkarzinom effektiv mittels metastasengezielter Radiatio behandelt werden [20].

Interessant ist v. a., dass eine Metastasektomie auch Auswirkungen auf die Symptome der Patienten haben kann. So besserte sich bei Patienten mit symptomatischen Metastasen die tumorassoziierten Symptome als auch der WHO Performance Score von 3,3 auf 2,1 (*p* = 0,005; [21]). Zusammenfassend profitieren Patienten v. a. bei singulären pulmonalen Metastasen von einer Resektion der Metastasen. Entscheidungen zur Metastasektomie sollten im Rahmen eines multidisziplinären Tumorboards getroffen werden.

## Neuartige Therapieansätze

Begleiterkrankungen von Patienten mit MIBC oder oligometastasiertem Blasenkarzinom können Kontraindikationen für eine RZ aber auch für eine systemische Chemotherapie oder Radiatio darstellen. Eine Kombinationstherapie mit Immun-Checkpunkt-Inhibitoren (ICI) und Strahlentherapie könnte durch die Rekrutierung von zytotoxischen T‑Zellen die Sensibilität für die Strahlentherapie erhöhen. Dies wiederum führt zu einer besseren Kontrolle des Primärtumors und resultiert in einem „Immungedächtnis“, welches sich auch systemisch auf potenzielle Metastasenbildung auswirkt [6].

Auch in der metastasengezielten Therapie wurde diese Kombination erfolgreich evaluiert. Kürzlich erhielten Patienten mit metastasiertem Blasenkarzinom entweder eine stereotaktische Bestrahlung der Metastasen gefolgt von einer Immuntherapie mit Pembrolizumab oder eine konkomitante Strahlen- und Immuntherapie. Die Gesamtansprechrate lag dabei bis zu > 40 %, wobei ein Therapieansprechen auch mit einem Rückgang der zirkulierenden Tumor-DNA (ctDNA) einherging [22].

Eine Kombinationstherapie mit ICI könnte die Sensibilität für die Strahlentherapie erhöhen

Das Ansprechen auf Therapien wird ebenfalls durch die histopathologischen Charakteristika des Blasenkarzinoms beeinflusst. Patienten, welche präoperativ eine Chemoradiatio erhielten, zeigten bessere Ansprechraten, wenn das Tumorgewebe einem genomisch instabilen oder plattenepithelial differenzierten Subtyp entsprach [23]. Eine weitere Phase-I/II-Studie mit Patienten, welche nicht für eine RZ geeignet waren, bezog die Expression von Her2/neu in die Therapieentscheidung mit ein. Die 62 Patienten erhielten Paclitaxel und tägliche Strahlentherapie. Wenn Her2/neu im Tumor hochreguliert war, erhielten diese Patienten zusätzlich den monoklonalen Antikörper Trastuzumab. Die Therapie beider Gruppen war effektiv bei einer hohen Rate an vollständig durchgeführten Therapien (60 % sowie 74 %) sowie moderater Toxizität (Nebenwirkungen in 35 und 30,4 % der Patienten; [24]).

## Fazit für die Praxis

- Bei der Oligometastasierung spricht man von einer Übergangsphase, in welcher ein Großteil der Tumorlast behandelbar ist und somit für den Patienten durch gezielte Therapie das Überleben verbessert werden kann.
- Patienten mit lokal fortgeschrittenem Urothelkarzinom der Blase sollten gemäß Leitlinien eine neoadjuvante Cisplatin-haltige systemische Chemotherapie und eine radikale Zystektomie (RZ) erhalten. Sprechen Begleiterkrankungen dagegen, ist eine trimodale Therapie (TMT) der RZ nicht unterlegen. Außerdem sollten neuere Therapiekonzepte wie die Immuntherapie bei Kontraindikationen für Cisplatin-haltige Therapie in Erwägung gezogen werden.
- Beim lymphogenen oder oligometastasierten Blasenkarzinom kann eine RZ zur Konsolidierung abhängig von der Ansprechrate auf die induktive Chemotherapie durchgeführt werden.
- In die Therapieentscheidung sollten neben dem Staging und Grading des Tumors auch die Komorbiditäten und der Performancestatus des Patienten hineinfließen.
- Der Stellenwert der adjuvanten Immunerhaltungstherapie in diesem Zusammenhang Situation ist noch unklar.
