# Erratum zu: Lokal fortgeschrittene oder oligometastasierte Blasenkarzinome – Stellenwert der Lokaltherapie von Primärtumor und Metastasen

**DOI:** 10.1007/s00120-022-01789-5

**Published:** 2022-03-01

**Authors:** Katharina Rebhan, Kilian M. Gust

**Affiliations:** Universitätsklinik für Urologie, Medizinische Universität Wien, Währinger Gürtel 18–20, 1090 Wien, Österreich

**Erratum zu:**

**Urologe 2021**

10.1007/s00120-021-01712-4

In diesem Artikel wurde der Name von Autor Katharina Rebhan fälschlicherweise als K. Rebhan angegeben. Der Originalartikel wurde korrigiert.

